# Supplementary material for: Genetic Diversity, Ochratoxin A and Fumonisin Profiles of Strains of Aspergillus Section Nigri Isolated from Dried Vine Fruits
Source: Toxins (Basel). 2020 Sep 14;12(9):592. doi: 10.3390/toxins12090592 (PMC7551007; doi:10.3390/toxins12090592)
Supplement: Supplementary file 1 [file toxins-12-00592-s001.pdf]

Supplementary Materials: Genetic diversity and Ochratoxin A profile of strains of *Aspergillus* section *Nigri* isolated from dried vine fruits

Petra Mikušová, Miroslav Caboň, Andrea Melichárková, Martin Urík, Alberto Ritieni and Marek Slovák

Table S1. List of reference strains and GenBank accession numbers used in the study. An Ex-type strain as defined in McNeill et al. ([1], Recommendation 8B).

Species	Ex-type	ITS	CaM	BenA
Aspergillus aculeatinus	CBS 121060	EU159211	EU159241	EU159220
Aspergillus aculeatus	CBS 172.66	EF661221	EF661148	HE577806
Aspergillus brasiliensis	CBS 101740	FJ629321	FN594543	FJ629272
Aspergillus brunneoviolaceus	CBS 621.78	AJ280003	EF661147	EF661105
Aspergillus carbonarius	CBS 111.26	EF661204	EF661167	EF661099
Aspergillus costaricaensis	CBS 115574	DQ900602	FN594545	FJ629277
Aspergillus ellipticus	CBS 482.65	EF661194	EF661170	EF661122
Aspergillus eucalypticola	CBS 122712	EU482439	EU482433	EU482435
Aspergillus floridensis	NRRL62478, ITEM14783	-	HE984429	HE984412
Aspergillus heteromorphus	CBS 117.55	EU821305	EF661169	EF661103
Aspergillus homomorphus	CBS 101889	EF166063	FN594549	AY820015
Aspergillus ibericus	NRLL 35644	EF661200	EF661163	EF661102
Aspergillus indologenus	CBS 114.80	aj280005	AM419750	AY585539
Aspergillus japonicus	CBS 114.51	AJ279985	FN594551	HE577804
Aspergillus labruscus	ITAL 22.223	KU708544	KT986008	KT986014
Aspergillus luchuensis	CBS 205.80	JX500081	JX500071	JX500062
Aspergillus neoniger	CBS 115656	FJ491682	FJ491700	FJ491691
Aspergillus niger	CBS 554.65	EF661186	EF661154	EF661089
Aspergillus piperis	CBS 112811	EU821316	EU163267	FJ629303
Aspergillus saccharolyticus	CBS 127449	HM853552	HM853554	HM853553
Aspergillus sclerotiicarbonarius	CBS 121057	EU159216	EU159235	EU159235
Aspergillus sclerotioniger	CBS 115572	DQ900606	FN594557	FJ629304
Aspergillus trinidadensis	NRRL 62479	-	HE984434	HE984420
Aspergillus tubingensis	NRRL 4875	EF661193	EF661151	EF661086
Aspergillus uvarum	CBS 121591	AM745757	AM745755	AM745751
Aspergillus vadensis	CBS 113365	AY585549	FN594560	AY585531
Aspergillus welwitschiae	CBS 139.54	FJ629340	KC480196	FJ629291

Table S2: Genotype assignments of isolated strains. GenBank accession numbers are indicated in the brackets.

<table>
<tr><th>Species</th><th>ITS Ribotype</th><th>CaM Haplotype</th><th>benA Haplotype</th><th>Concatenated ITS/CaM/benA Genotype</th><th>Isolated Strains</th></tr>
<tr><td rowspan="2">A. carbonarius</td><td rowspan="2">ITS_IV (MK046883)</td><td rowspan="2">CaM_IX (MK046876)</td><td>benA_I (MT166308)</td><td>Concat_I</td><td>G_187</td></tr>
<tr><td>benA_II (MT166309)</td><td>Concat_II</td><td>G_191</td></tr>
</table>

A. luchuensis	ITS_I (MK046882)	CaM_VII (MK046871)	benA_III (MT166310)	Concat_III	G_062
		CaM_VI (MK046872)	benA_IV (MT166311)	Concat_IV	G_198
			benA_V (MT166312)	Concat_V	G_201 G_204
			benA_VI (MT166313)	Concat_VI	G_202
		CaM_VIII (MK046879)	benA_VII (MT166314)	Concat_VII	G_199
A. tubingensis		CaM_III (MK046873)	benA_VIII (MT166315)	Concat_VIII	G_132
			benA_IX (MT166316)	Concat_IX	G_172
		CaM_I (MK046874)	benA_X (MT166317)	Concat_X	G_184
			benA_XI (MT166318)	Concat_XI	G_183
			benA_XII (MT166319)	Concat_XII	G_168
			benA_XIII (MT166320)	Concat_XIII	G_178 G_188 G_211
			benA_XIV (MT166321)	Concat_XIV	G_176
			benA_XV (MT166322)	Concat_XV	G_182
			benA_XVI* (MT166323)	Concat_XVI	G_190
			benA_XVII (MT166324)	Concat_XVII	G_174 G_180
		CaM_II (MK046870)		Concat_XVIII	G_045 G_160
			benA_XVI* (MT166323)	Concat_XIX	G_061
		CaM_IV (MK046875)	benA_XVIII (MT166325)	Concat_XX	G_171
		CaM_V (MK046877)	benA_XIX (MT166326)	Concat_XXI	G_192
A. aff. welwitschiae	ITS_III (MK046881)	CaM_XI (MK046869)	benA_XX (MT166327)	Concat_XXII	G_036
			benA_XXI (MT166328)	Concat_XXIII	G_166
A. welwitschiae	ITS_II (MK046880)	CaM_XII (MK046878)	benA_XXII (MT166329)	Concat_XXIV	G_196

A. niger	*A.*	CaM_X (MK046868)	benA_XXIII (MT166330)	Concat_XXV	G_033<br>G_209<br>G_210
			benA_XXIV (MT166331)	Concat_XXVI	G_050

* Strains G_061 and G_190 represent identical genotype benA_XVI. Strains G_085, G_131, G_189, G_212, G_203 were not assigned to genotypes due to missing data and their position is resolved in maximum likelihood analysis.

Table S3. The list of strains selected for individual analyses for toxigenic ability using HPLC, Abbreviations: b.d.l. = below detection limit, FB2 = fumonisin B2, FB1 = fumonisin B1.

Asergillus sect. Nigri	Strain Code	Level of FB1 (μg/kg)	Level of FB2 (μg/kg)	Level of OTA (μg/kg)
A. carbonarius	G_187	b.d.l.	b.d.l.	4381,911873
A. carbonarius	G_191	b.d.l.	b.d.l.	2477,256
A. luchuensis	G_199	b.d.l.	b.d.l.	b.d.l.
A. luchuensis	G_202	b.d.l.	b.d.l.	b.d.l.
A. luchuensis	G_204	b.d.l.	b.d.l.	b.d.l.
A. luchuensis	G_201	b.d.l.	b.d.l.	b.d.l.
A. luchuensis	G_198	b.d.l.	b.d.l.	b.d.l.
A. luchuensis	G_062	b.d.l.	b.d.l.	b.d.l.
A. niger	G_033	1213,375	11118,651	b.d.l.
A. niger	G_209	510,889	5509,260	b.d.l.
A. niger	G_210	535,653	5568,915	b.d.l.
A. niger	G_050	611,370	7413,953	b.d.l.
A. welwitschiae	G_036	b.d.l.	b.d.l.	b.d.l.
A. welwitschiae	G_166	b.d.l.	b.d.l.	b.d.l.
A. welwitschiae	G_196	b.d.l.	b.d.l.	b.d.l.
A. tubingensis	G_178	b.d.l.	b.d.l.	b.d.l.
A. tubingensis	G_180	b.d.l.	b.d.l.	b.d.l.
A. tubingensis	G_132	b.d.l.	b.d.l.	b.d.l.
A. tubingensis	G_160	b.d.l.	b.d.l.	b.d.l.
A. tubingensis	G_171	b.d.l.	b.d.l.	b.d.l.

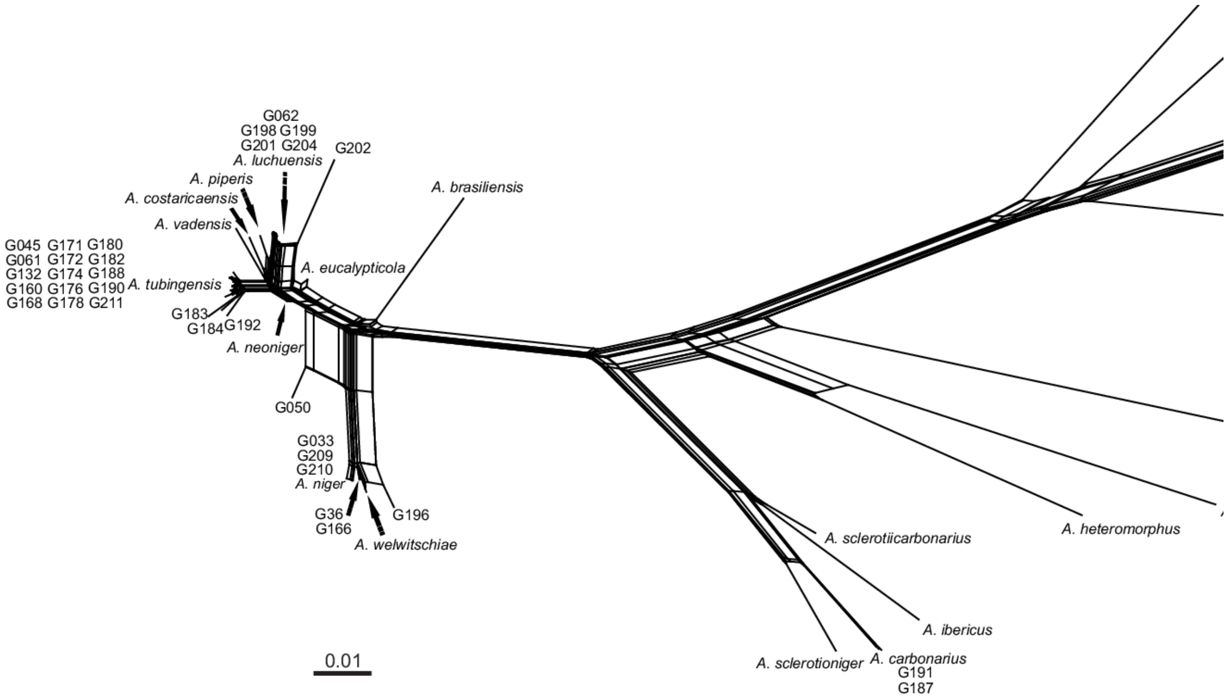

Figure S1. The neighbour-net diagram as inferred from the concatenated dataset composed of ITS region, calmodulin and betatubulin genes. Only biseriate species (groups) of black aspergilli are shown. All species accessions from the *Aspergillus* section *Nigri* are represented by their type strains (Table S1). For details on accession codes of our strains see Table S2.

Reference

1. McNeill, J., Barrie, F. R., Buck, W. R., Demoulin, V., Greuter, W., Hawksworth, D. L., Herendeen, P. S., Knapp, S., Marhold, K. & other authors (2012). International code of Nomenclature for algae, fungi, and plants (Melbourne Code), adopted by the Eighteenth International Botanical Congress Melbourne, Australia; July 2011. Koeltz Scientific Books: Oberreifenberg, Germany. 2012. ISBN: 978-3-87429-425-6
